# Experiences of people with complex mental health difficulties accessing help from primary care services: a qualitative interview study

**DOI:** 10.1186/s12875-026-03322-5

**Published:** 2026-05-06

**Authors:** Rachel Instone, Ada Achinanya, Chris Burton, Eleni Chambers, Michelle Horspool, Tom Isherwood, Philip Oliver, Vyv Huddy

**Affiliations:** 1 Tees, Esk And Wear Valleys NHS Foundation Trust, Darlington, UK; 2 University of Sheffield, Sheffield, UK; 3 Sheffield, UK; 4 Sheffield Health Partnership University NHS Foundation Trust, Sheffield, UK; 5 University of Leeds, Leeds, UK

**Keywords:** Complex mental health difficulties, Primary care, Access, Thematic analysis

## Abstract

**Background:**

Primary care is often the first point of contact for adults with complex mental health difficulties, yet many experience persistent barriers to accessing timely and appropriate support. Despite policy commitments to integrated and equitable care, there remains a need to better understand how individuals make sense of distress and navigate services that may struggle to meet complex needs. This study aimed to better understand the experiences of adults with complex mental health difficulties as they access primary care services. It sought to further understand how they made sense of their difficulties accessing care and their psychological distress.

**Method:**

This study used a qualitative design with reflexive thematic analysis. Nineteen participants with complex mental health difficulties were interviewed using a semi-structured schedule. They were recruited via their GP practices.

**Results:**

Three themes were identified: participants’ desire to understand why they felt the way they did, the mental health condition itself creating barriers to care, and the precarity of self-management and help. An overarching theme of “Contradictions” was evident across all three themes. “Contradictions” included professionals talking of connecting but then distancing, help being offered but never arriving, trauma being present but ignored, and diagnosis getting in the way of receiving care.

**Conclusions:**

Overall, the study provided further evidence that those with complex mental health difficulties are currently underserved in the NHS. Novel insight into their health literacy questioned the assumptions that those with complex mental health difficulties lack understanding; rather, it provides evidence that services need to collaborate effectively with service users to enable better communication.

**Supplementary Information:**

The online version contains supplementary material available at 10.1186/s12875-026-03322-5.

## Introduction

Complex mental health difficulties (CMHD) is an umbrella term for people who live with multiple sources of distress. The term was suggested in the consensus statement from MIND [1], which addressed the problematic label of “personality disorder”. The statement argued that the difficulties some people experienced were better understood as interrelated social, psychological and developmental factors [1]. People with complex mental health difficulties may meet criteria for, or have been given, diagnoses such as personality disorder, complex trauma, or persistent depressive conditions (historically referred to as dysthymia). “Complexity” is also described in the Community Mental Health Framework for Adults and Older Adults [2], and includes neurodiversity.

Primary care is intended to support individuals with CMHD. People with common mental health difficulties like anxiety and depression can be supported by NHS Talking Therapies, originally known as Improving Access to Psychological Therapies (IAPT). Expanded services to treat a broader range of issues and treatment offerings indicate the potential for more widely accessible psychological therapy [3]. However, whilst they offer interventions to address the needs of those with more complex mental health difficulties, there is lack of evidence on the appropriateness and effectiveness of interventions offered [4]. Furthermore, specialist mental health services frequently reject referrals for individuals with CMHD from General Practitioners (GPs) as they do not meet their acceptance criteria, leaving people with a high need for care without adequate support [5]. Although “complex mental health difficulties” (CMHD) is not a formally coded category in primary care, related diagnoses are under-recorded in electronic health records. United Kingdom primary care data indicate that only around 1–1.5% of patients have a coded diagnosis of personality disorder, despite substantially higher community prevalence estimates [6]. For example, previous research has estimated that approximately one quarter of individuals presenting to primary care with mental health difficulties meet criteria for personality disorder [7]. This suggests that routinely recorded data underestimate the scale of complex and enduring mental health needs seen in primary care. Furthermore, the concept of complex mental health difficulties explicitly recognises that such presentations are not reducible to personality disorder alone and may instead be characterised by constellations of overlapping conditions.

Adults with CMHD often face structural barriers that impede access to care. Social determinants such as disadvantage, discrimination, social isolation, housing instability and financial pressures can make attending appointments difficult, sometimes resulting in premature discharge from services [8–12]. Furthermore, these factors impact an individual’s ability to understand their health and health needs is dependent on their level of disadvantage, insight into their condition and understanding of good mental and physical health [13–15]. These structural challenges intersect with stigma: diagnostic labels under the CMHD umbrella, including personality disorder, are highly stigmatised. Clinician biases, discriminatory practices and dominance of the biomedical model can negatively affect engagement, self-esteem and quality of life [16–18]. Neurodevelopmental differences can compound these experiences with individuals reporting that traits are frequently misunderstood or misattributed to other mental health conditions [19–22]. Together these social and structural factors create multiple overlapping barriers to accessing appropriate support for adults with CMHD.

The Community Mental Health Framework for Adults and Older Adults sets out new expectations for primary care to meet the needs of individuals with CMHD [2]. Its definition of complexity includes people with previous negative experiences of services, neurodevelopmental conditions, substance use problems, age-related frailty and physical health difficulties which frequently co-occur and complicate care provision. Despite these policy aims, there remains limited evidence on how adults with CMHD experience accessing support within primary care. Existing qualitative research in primary care has largely focused on specific diagnostic groups, such as personality disorder or chronic depression, rather than examining the broader population of people living with complex mental health difficulties [23].

The UNSEEN (Understanding Services for Complex Mental Health Difficulties) study addressed this gap using a mixed methods approach to examine how general practices identify and support people with CMHD [24]. The current study conducted a secondary analysis of these data to report the lived experiences of people with CMHD to enable a more in-depth and theoretically informed exploration of patient experiences of care than was possible within the original mixed methods analysis. This current investigation was more explicitly informed by consideration of the role of the social determinants of health in shaping experiences of distress and service access.

This study aimed to generate a deeper understanding of the experiences of adults with CMHD in relation to accessing care. Specifically, it addressed the following questions: Firstly, how do adults with CMHD describe and understand their difficulties? Secondly, how do they experience accessing primary care and other sources of support?

## Method

### Patient and public involvement

Three individuals with lived experience of complex mental health difficulties and use of relevant services were involved in shaping the overarching study and its design. Advice was given on participant recruitment. In addition, they advised on ethical issues, gave feedback on study documents including the development of the information sheets, topic guides, with one of the group (author EC) reviewing the findings to ensure coherence and credibility. Author EC has been involved in the process of write-up and dissemination.

### Participants

This study is part of a wider project which aims to provide a framework for GPs to better identify patients under their care who have CMHD. Funding was approved by the National Institute for Health Research through the Research for Patient Benefit programme (NIHR203473), and Health Research Authority approval (REC reference: 22/EM/0099) was granted for our study.

Participants were recruited via general practices based in South Yorkshire, UK through the NIHR Clinical Research Network (CRN). There was a purposeful aim to focus on practices in areas of relatively high deprivation (practices in the most deprived 40% of the English Index of Multiple Deprivation). The GP practices that agreed to participate in the study were given information packs with instructions for searching their electronic databases. General practice staff undertook the electronic searches using the search strategy developed and piloted by the research team. The search strategy included codes for “personality disorder”, “complex PTSD”, and “dysthymia”. Once potential participants were identified, the practice GP was asked to manually review the list to ensure the patients identified were suitable for an interview based on the inclusion and exclusion criteria. The inclusion criteria were adults aged over 18, who were identified by their GP as having complex mental health difficulties, including neurodiversity, were able to access online video conferencing software or telephone and, finally, where their mental health considered stable enough to participate in the study as determined by their GP. In total, 19 patient participants were recruited using purposive sampling and interviewed from seven practices, and all the gathered data were used in the analysis. This breadth of data allowed the researcher to reach well-supported themes drawn across the sample [25–27]. Recruitment continued until no substantially new information was emerging from the interviews (data saturation) [28], and the research team judged that additional interviews were unlikely to generate new insights relevant to the research question. Participants were aged 20–79; there were fourteen women and five men. Although recruitment had been targeted at general practices in diverse ethnic areas, all participants were White British apart from one South Asian participant. See Table 1 for further details.

**Table 1:** Participant Demographics

Participant	Age	Gender	Ethnicity	Diagnoses
1	60–79	F	White British	Complex Emotional Needs
2	20–39	F	White British	Persistent Depression
3	60–79	F	White British	Autism Spectrum Condition
4	40–59	F	White British	Complex Emotional Needs
5	20–39	M	White British	Psychosis
6	40–59	F	White British	Bipolar Disorder, Anxiety Disorder
7	20–39	F	White British	Dissociative Identity Disorder
8	20–39	F	White British	Anxiety Disorder, Complex Emotional Needs
9	40–59	F	White British	Bipolar Disorder, Anxiety Disorder, Complex Emotional Needs
10	40–59	M	White British	Persistent Depressive Disorder, Complex Emotional Needs, Anxiety
11	40–59	M	South Asian	Persistent Depressive Disorder, Complex Emotional Needs, PTSD
12	40–59	F	White British	Complex Emotional Needs, Depression, Anxiety Disorder
13	40–59	M	White British	Dissociative Personality Disorder, Schizophrenia
14	40–59	F	White British	Complex Emotional Needs, Depression, Childhood trauma
15	60–79	M	White British	Post Traumatic Stress Disorder, Depression
16	20–39	F	White British	Complex Emotional Needs, Post Traumatic Stress Disorder, Attention Deficit Hyperactivity Disorder, Autism Spectrum Condition
17	20–39	F	White British	Complex Emotional Needs, Depression, Autism Spectrum Condition
18	20–39	F	White British	Complex Emotional Needs, complex Post Traumatic Stress Disorder, prenatal depression
19	40–59	F	White British	Complex Emotional Needs, Depression, Anxiety

### Procedure

#### Interview schedule

A semi-structured interview schedule was developed for the UNSEEN study [24] and is available in supplementary materials. This was created in collaboration with three people with lived experience of CMHD who also consulted on the wider project and followed the guidance for reflexive thematic analysis [27, 29]. The interview topic guide covered perceptions of complex mental health difficulties, experiences of accessing and collaborating with GPs and other mental health services that interact with primary care, and the impact of difficulties on family and support networks. Semi-structured interviews were conducted online, recorded and transcribed verbatim for analysis. All interviews were conducted by AA between January and June 2023 and lasted between 60 and 120 min.

### Data analysis

Reflexive thematic analysis provides a flexible, thorough and reflexive approach to the data [25, 29]. Reflexive thematic analysis allows for an analysis across a data set, providing insights and interpretations and going beyond the merely descriptive. The development of themes is an iterative process that allows the researcher to bring layered interpretations to the experiences, thoughts and beliefs of the participants. It brings a depth of understanding and allows for an inductive approach to answering research questions [26]. Widely used in health and psychological research, reflexive thematic analysis has been executed in various ways. This study used the well-documented guidelines of Braun and Clarke [27, 29].

Data analysis was conducted by RI. who kept a reflective journal to document her thoughts on the transcripts from the first reading and listening as the analysis developed. RI identifies as a white middle-aged cisgender female who has lived experience of accessing diagnostic and other services for family members relating to learning disability and neurodiversity. The transcripts were read, and recordings were listened to several times, allowing the researcher to immerse herself in the words and expressions of the participants. Initial notes were made to capture early interpretations, reflections, and responses to the narratives. These notes formed the starting point for developing codes and exploring patterns across the dataset. The dataset was then coded in NVivo (version 14), with codes applied to segments of data relevant to the research questions. Themes were iteratively reviewed and refined by examining their coherence in relation to both coded extracts and the dataset as a whole. This process involved developing and revising a thematic map and refining the definitions and boundaries of themes.

### Rigour and quality procedures

To support analytic rigour, elements of the analysis were discussed with a second researcher (TI) and co-supervisors experienced in qualitative methods and clinical practice. Two transcripts were independently reviewed by TI during the analytic process. Although primary coding and theme development were undertaken by the first author, transcripts, codes, and developing interpretations were reviewed and discussed with co-supervisors, who provided written and verbal feedback on the analytic decisions and thematic structure. These discussions focused on the coherence and boundaries of themes, the relationships between themes, and alternative interpretations of participants’ accounts. The developing thematic framework was also discussed with an expert-by-experience involved in the study design (EC), who was familiar with the dataset. This discussion supported reflexive consideration of how the interpretations resonated with lived experience perspectives.

### Reflexivity

Reflexive thematic analysis recognises that researchers’ backgrounds shape interpretation of the data. The researchers involved directly involved in the analyses reported here (RI, VH and TI) are clinical psychologists with experience working with people with complex mental health difficulties across primary care, secondary mental health, and learning disability services. This professional background provided familiarity with the contexts participants described but also brought prior assumptions about barriers to care and help-seeking. These assumptions were considered throughout the analytic process through reflective journaling and discussions in supervision. The wider UNSEEN research team, including experienced practising GPs (PO, CB) and a qualitative researcher (AA), also reviewed and commented on the developing thematic account during manuscript preparation.

## Results

**Fig. 1:**
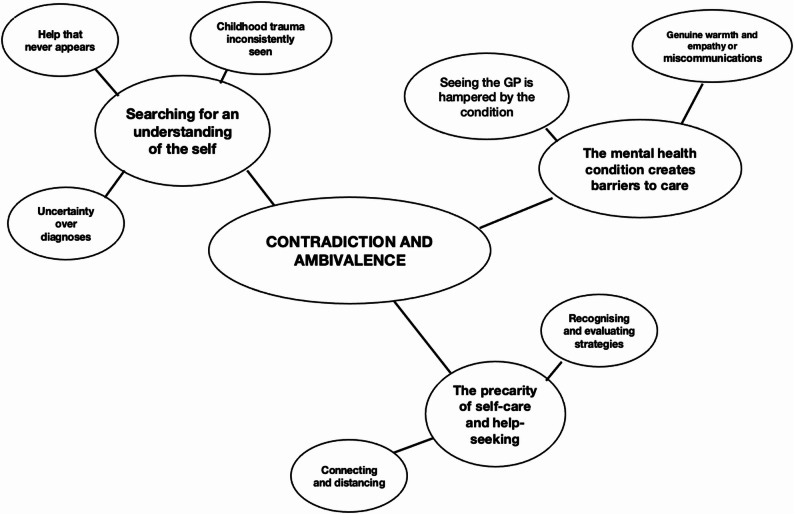
Thematic Map

Reflexive thematic analysis was used on the full dataset of 19 participants. Participants, all living in areas of socioeconomic deprivation, described a range of challenges navigating primary care services. See Fig. 1 for how the themes were organised and related to each other. Across participants’ accounts, tensions and ambivalences were frequently evident. This cross-cutting pattern of contradiction appeared across several themes and is returned to at the end of the results section.

The first theme, “Searching for an understanding of the self,” captures participants’ efforts to make sense of their mental health difficulties and past experiences. Sub-themes of “uncertainty over diagnoses,” “childhood trauma as inconsistently seen,” and “help that never appears” describe participants’ attempts to obtain clarity and validation from services while encountering fragmented or limited explanations of their difficulties.

The second theme, “The mental health condition creates barriers to care,” describes how participants’ symptoms and experiences shaped their interactions with services and help-seeking. Sub-themes of “seeing the GP is hampered by the condition” and “genuine warmth and empathy or miscommunications” illustrate how encounters with clinicians could feel either supportive or alienating.

The third theme, “The precarity of self-care and help-seeking,” reflects participants’ efforts to manage distress while navigating relationships and sources of support. Sub-themes of “recognising and evaluating strategies” and “connecting and yet distancing” highlight the careful and ongoing negotiation involved in seeking help and maintaining connections with others.

### Theme 1: Searching for an understanding of the self

#### Uncertainty over diagnoses

Participants described uncertainty about their diagnoses, both in their own understanding and in how they perceived clinicians (including GPs) to interpret their difficulties. At times, participants felt that responsibility for identifying a possible diagnosis had been placed on them, rather than being collaboratively explored with the clinician.

*“[she] told me to go off and look at MIND and think about what I thought I had*,* which I thought was very odd; I’m not a doctor*,* I’m not a psychiatrist*,* I don’t have any knowledge … So I went back*,* and she said ‘well what do you think?’” P18*.

Other participants had experienced delays or their diagnoses changing as their understanding of conditions deepened or were challenged. Some participants who were later diagnosed with autism or ADHD, recalled being told by clinicians that they could not have these conditions because they were female, or were managing life “too well”. Participants described how this type of contradictory advice created uncertainty and confusion. There was also a sense of urgency and frustration in this theme as participants attempted to find a cause for their distress.

*“I drive myself crackers to be honest. It’s like I’m constantly searching for an answer that’s going to fix my brain.” P6*.

Participants expressed that it was important to reach this understanding with the help of a professional in the hope that this would give them certainty about their condition. This would then, it was hoped, lead to appropriate support. Participants described feeling satisfied when their diagnosis felt heard and understood; there was a sense of collaboration rather than just being given a diagnosis.

*“So from there*,* I’d been able to say to GPs ‘BPD’*,* and then it’s like ‘oh right*,* OK*,* we know what that means’*,* you’re not just a little bit depressed*,* OK.” P4*.

#### Childhood trauma inconsistently seen

Many participants described spending a lot of time self-questioning and reflecting when seeking answers and understanding of their conditions. There was an acknowledgement that their early life experiences and significant events had influenced their mental well-being, but this was not always referenced by professionals.

*“you’re the first one to say ‘look … could be childhood trauma’*,* like I’m not being rude because you’re the first one to say it*,* but I figured that out for myself a long time ago” P5*.

This highlights how participants described feeling they had a strong understanding of the origins of their difficulties, yet this personal insight was often unacknowledged or overlooked in clinical consultations. This created a further contradiction: while participants believed it was the clinician’s role to provide explanations and validation, they also experienced themselves as holding valuable knowledge that was not recognised within that professional framework.

#### Help that never appears

This sub-theme is about the difficulty that participants described in accessing appropriate services once they had a diagnosis or it was recognised that they might need some or more help. It speaks to the sense of frustration trying to navigate mental health services, the opaque nature of where to go and how to access help. They spoke of being referred for secondary services, but the support either never materialising or being so short-lived that they only had time to disclose their most significant traumas before their limited sessions ended:

*“It’s like dangling a carrot in front of a donkey*,* so it looks on paper that you’re doing something and… keeping my hopes that I’m getting help but it never appears” P6*.

Participants described how inflexible services, contradictory messaging and, at times, complete lack of provision left them feeling abandoned. The lack of services to meet their needs was present throughout the participants’ interviews. Primary care services referred them to secondary services but then they perceived “rejection”, compounding the feelings of being “untreatable”. Participants felt not only the stigma associated with their diagnosis, as reflected in staff reactions, but they also came to understand themselves as unworthy of care.

*“IAPT … looked at the words ‘personality disorder’ and ‘PTSD’ and went ‘no*,* no*,* no*,* we can’t deal with her*,* like*,* we’re not equipped to help her or provide support for her’ … But there’s nothing else. There’s nowhere else to go.” P16*.

### Theme 2: The mental health condition itself creates barriers to care

This second theme relates to how participants mental health manifested, which often precluded them from accessing care. Participants described feelings of shame about their difficulties, which sometimes prevented them from seeking help from services:

*“I used to drink a lot because it used to help mask my feelings. I was ashamed to go to the doctor’s because I thought somebody like me shouldn’t be doing*,* you know*,* the drinking.” P1*.

Participants spoke of unhelpful administrative staff and inflexible booking systems that made accessing care for their complex problems more difficult. For example, one participant described the challenge of navigating appointment systems when already struggling with severe distress generated by complex mental health problems:

*“It’s so difficult to get GP appointments and when you really are at rock bottom with your mental health and you are struggling to even get out of bed or brush your teeth… the prospect of having to … call at 8am” P16*.

#### Seeing the GP is hampered by the condition

This sub-theme reflects the difficulties that participants had in accessing their GP due to their condition. This theme shows how not being able to reach the GP as the “*first line of treatment*” *(P16)* increases the risk of emergency or crisis services being needed. Participants spoke of their GP not recognising when they needed to seek help. They then described that they were only offered help when distress became extremely heightened and visible. Even then, the help that was offered was often perceived to be distant or wholly inadequate.

“*Unless you do some harm*,* either to yourself physically*,* or to someone else*,* nobody seems to really care or actually be that bothered.” P13*.

There was also the sense in this theme that participants could monitor changes in their emotional states, which, if recognised by GPs and secondary mental health clinicians, would be one way to provide person-centred, attuned care when it was needed.

*“I have severe flashbacks*,* especially now building up to the anniversary*,* and I know it’s going to happen because I can smell grass when I’m asleep*,* and then I wake up*,* and then it starts. And it’s not pleasant at all…” P15*.

#### Genuine warmth and empathy or miscommunications

This sub-theme reflects how participants described the ways their distress was responded to. They reported both dismissive and demeaning encounters, as well as positive encounters with GPs and mental health professionals. Regular check-ins with a familiar GP were seen as being beneficial and helped to establish trust. Continuity of care was key for all the participants, as this fostered a trusting relationship with a GP allowing for true collaborative care.

*“I have a brilliant GP who knows me and understand me and he’s a lifeline*,* he’s saved me so many times*,* more than he knows*,* because he’s always been there and always listened and taken care of me.” P15*.

The benefit of developing that trusting relationship with the participant was gaining a more holistic understanding of their needs, something which the participants acknowledged as being important for their treatment. Participants described how being excluded from decision-making was unhelpful. Instead, they wanted honest and open communication that would enable them to make informed choices about treatment options.

*“I’d rather someone just be straight-up honest with me because I’m an adult. Treat me like an adult*,* yeah.” P8*.

### Theme 3: The precarity of self-care versus help-seeking

This last theme reflects the varied ways that the participants found to help themselves in their distress, which varied between seeking support from others and self-reliance. All participants described finding ways to cope with the challenges in their lives, juggling family responsibilities, work and the distress resulting from their untreated conditions. For some, these coping strategies included behaviours that were difficult to understand at the time, such as self-harm:

*“Obviously I started self-harming and at that point I didn’t really know why I was doing it*,* other than it helped me*,* the physical pain was a distraction from everything going on in my head that I couldn’t work out.” P16*.

Some participants recognised the need for more manageable lives and made changes accordingly. Others described developing personal strategies to cope with distress and intrusive thoughts:

*“you have to come up with systems to help yourself*,* like … I’ve got suicidal thoughts … instead of being lethal with it*,* I starve myself*,* so it gives you time to think about it … So far*,* touch wood*,* is a tried and tested method for myself.” P5*.

Others described drawing on various sources of inspiration, including from friends and online resources. Overall, participants expressed a strong determination to survive and manage their distress, which may represent a resource that could be recognised and explored in consultations with clinicians.

#### Recognising and evaluating strategies

Building on the previous sub-theme of precarity, participants described evaluating their own coping mechanisms and developing insights into their usefulness. In some cases, this involved recognising that these strategies were unhelpful, prompting them to seek professional help. One participant used the metaphor of repairing a burst pipe with tape to describe how their current coping strategies felt like temporary fixes rather than lasting solutions:

*“so currently I’ve asked to be on a waiting list to do that to help with better coping skills*,* because a lot of what I’m doing is bodged…if you’ve got a burst pipe*,* rather than replacing the pipe you’ll get a load of tape and just tape it over it.” P5.*

However, some participants were critical of treatments that were offered by services they were referred to by GPs, whether that was medication or talking therapies. These accounts were also reflected within this theme. In particular, participants felt that the therapy they were referred to lacked flexibility:

*“They seem to have a one size fits all approach to therapy. It’s been mainly told what to do.”* P10.

Some people described engaging passively with the group therapy treatment they were offered after referral by their GP.

*“I just sit there with a cup of tea and a biscuit and sometimes I just do colouring or I just do a stream of consciousness in my notebook and it’s fairly cathartic for me.” P17*.

Another participant described not understanding how the therapy they were referred to was intended to work and felt that the approach lacked person-centred care or genuine collaboration

*“It were just like ‘Do that’ and ‘Tap your wrist’ and ‘Tap your nose’ and ‘Tap your head’. No thanks. … I just told them straight … that’s not for me. That’s definitely not for me.” P10*.

Thus, these experiences capture a process of recognising their coping strategies, evaluated these as ineffective, and then sought professional help. However, when that professional help they were referred to was received, it was unsatisfactory and left participants feeling unsupported:

*“He always turned round and said to me ‘Mum*,* you need proper help’ and I says ‘well I’m not getting it am I’” P9.*

#### Connecting and yet distancing

A second sub-theme is about the tension of wanting to share their experiences with friends and family and worrying that doing so might cause harm. Participants spoke of trying to shield family from some aspects of their distress, both to protect them and to protect themselves. However, there were accounts of mutual learning that enabled more supportive relationships.

*“[My family] have learnt over the years*,* as I have I suppose. So now we’ve found a way for them to be able to support me.” P11*.

For others, there was no close relationship to provide support. Others described needing to carefully manage close relationships that could, at times, be detrimental to their well-being. There was a recognition that even though there were strong relationships, these were not without their challenges.

*“My mum*,* yeah*,* we’re quite close*,* but I try and take it arm’s length when she’s in a bad place.” P8*.

Supportive partners and friends provided consistency and positive regard, which gave participants a sense of safety. Some participants had long-serving friends or loved ones who they felt understood them and provided support, and there was an unspoken sense that they were lucky in this.

### Cross-cutting theme: contradiction and ambivalence

Across the themes, a cross-cutting pattern of contradiction and ambivalence shaped participants’ accounts. Participants often expressed clear ideas about what they needed from services while simultaneously feeling misunderstood or unseen by clinicians. Services were described as intended to offer responsive, tailored support, yet for many these expectations were not realised, contributing to disillusionment and self-doubt. Similar tensions appeared in relationships with loved ones, where a desire to share distress coexisted with concerns about burdening others.

These contradictions were evident across themes. In “Searching for an understanding of the self,” participants described uncertainty about diagnoses and attempts to make sense of past and present experiences, often encountering limited clarity or continuity of care. In “The mental health condition creates barriers to care,” participants recognised the need for support but described how their symptoms hindered help-seeking. Finally, “The precarity of self-care and help-seeking” illustrated how participants oscillated between self-reliance and the wish for connection, navigating support while feeling caught between engagement and withdrawal.

## Discussion

This research aimed to give voice to an often unseen and unheard group of people living with CMHD. Findings showed that this group faces many barriers to accessing primary care and other sources of support. However, they also reveal a resourcefulness and awareness of their difficult circumstances which is less frequently acknowledged by services and clinicians.

The overall findings were reflected within the overarching theme of “contradiction”. There are numerous reports of people with CMHD experiencing contradictory or mixed messages from services—such as being heard yet dismissed or offered care that feels coercive—undermining trust and reinforcing insecurity. For example, individuals with complex emotional needs described perceiving that their trauma histories were minimised, with participants expressing the view that the burden was on them to “educate” care providers [30]. In inpatient settings, involuntary admission has been framed by clinicians as protective but experienced by patients as coercive, a dual message that complicates the therapeutic relationship [31, 32]. Similarly, people receiving inpatient care also describe concurrently feeling both safety and shame during their admission because they perceived staff interacting with them erratically either as a unique individual or feeling positioned just as ‘a disorder’ [33]. From an attachment perspective, such inconsistencies may replicate early experiences of relational unpredictability, reinforcing attachment insecurities. Berry et al. found that staff members’ own attachment styles—particularly those high in avoidance or anxiety—were linked to weaker therapeutic relationships and reduced psychological mindedness, suggesting that caregivers’ emotional frameworks may contribute to how mixed messages are conveyed [34]. These tensions are also reflected in the wider primary care literature. A recent scoping review identified qualitative studies describing similar experiences of mixed or ambiguous signals within care, particularly in research focusing on personality disorder. However, few studies have examined these dynamics across the broader population of people with complex mental health difficulties [23].

The first theme was “finding and not finding an understanding of the self”. This spoke to the determination with which the participants sought understanding of their condition and wanted to know how they could be supported. The findings echoed previous work which has shown diagnosis can be a positive or a negative experience depending on how the process of diagnosis unfolds [35]. Participants expressed varying reactions to being given a diagnosis with some being relieved to have this. However, this study echoed previous research on the stigma of CMHD, and the poor attitudes of staff were still perceived to be present in services [30, 36].

Qualitative studies conducted in primary care settings have reported similar challenges for people with complex emotional needs, particularly around diagnosis, stigma, and navigating access to care. However, much of this literature has focused on specific diagnostic groups such as personality disorder, with fewer studies exploring these experiences across the broader population of people living with complex mental health difficulties [23]. The need for an understanding of their distress was universal across participants. Participants described researching their symptoms and persevering when services were hard to access. For some, finding a recognised diagnosis was important for some to understand themselves and their sense of place in society, which is consistent with social identity theory [37] and echoes qualitative work exploring service users’ experiences of personality disorder diagnosis [38]. However, the consequences of this were varied, with some experiencing stigma, which in turn was internalised and led to feelings of shame. Similar tensions around diagnostic labels and stigma have also been reported in primary care psychological therapy services [4]. This sense expressed of being “abandoned by services” resonates with the work on structural stigma. These findings align with work on narrative identity and collaborative meaning making between the clinician and service user during diagnostic conversations [39, 40]. The sense of abandonment expressed by participants can be viewed with an attachment lens, where difficulties in trusting services and ambivalence towards help seeking can be linked to earlier experiences with services [41].

The second theme, “the mental health condition itself creates barriers to care,” spoke to the effort required by participants to navigate their way into the system. Accessing GPs relied on participants having the capability and resources to call for help at a specific time in the morning with no flexibility of access. Difficulties navigating primary care access systems have also been noted in qualitative studies of chronic depression in general practice [42]. These findings suggest that the complexity of people’s lives is not always considered by services with too much required of the service user to access the care they need. Many of the participants found expressing their distress to clinicians overwhelming, and validation was often lacking. Challenges communicating distress to clinicians have also been described in studies of prescribing encounters for people with personality disorder in primary care [43]. Responses such as hypervigilance, shame, and avoidance may also shape how people engage with support services [44], and may explain why some may struggle within rigid or standardised care systems that can be difficult to navigate [45]. It highlights the importance of flexible, person-centred, and multi-agency approaches—particularly for people living with complex mental health difficulties (CMHD). Services that foster an understanding of emotions and distress as culturally constructed and experienced relationally may help to guide more collaborative conversations with service users [46]. As many participants had experienced trauma, an awareness of how to engage them in collaborative help-seeking is important for all clinicians, particularly those in primary care.

The final theme was the “precarity of self-care and help-seeking”. This theme encapsulated the sense that participants understood their needs but, at times, lacked confidence in their own insight as to why their mental health might be suffering. This finding brings a novel nuance to the idea of health literacy [13], which assumes that a knowledge deficit precludes people from accessing care rather than structural and relational barriers. The participants described understanding the role of social determinants of their distress [8, 47]. Similar findings have been reported in qualitative research exploring persistent depression in primary care populations [48]). However, when trying to engage in care-seeking, participants in the current study reported that they were not supported to share their insight that a more holistic approach would be beneficial. The Community Mental Health Framework recognises the importance of healthy communities with good housing and amenities [2]. Acknowledging the inequalities around access to housing, financial security, and healthy spaces destigmatises those suffering psychological distress by externalising the sources of it. This study supports previous evidence that has highlighted the need for longer appointments with GPs and continuity of care so a strong alliance between clinician and service user can be built [18, 43].

### Strengths and limitations

As a purposefully sampled study, GPs were successful in enabling the recruitment of 19 participants with diverse requirements that fall under the umbrella of the term of CMHD, who provided rich and detailed accounts of their experiences to inform the themes. Recruitment across multiple GP practices enabled a geographically distributed sample and supported the inclusion of participants with a range of experiences of services. As participants were recruited from practices serving areas of socioeconomic deprivation, these findings may particularly reflect the experiences of people living in deprived communities. Another strength is the design of the study, which involved people with lived experience of CMHD. The results were discussed with one of them to aid with the quality of the output and the legitimacy of the findings.

A limitation is that although recruitment was targeted to GP practices with ethnically diverse populations, this did not translate to a diversity in the ethnicity of the participants or their gender. Almost all participants identified as White British, with only one participant from a minority ethnic background. The sample was predominantly female, which may reflect gender differences in help-seeking and participation in mental health research. As a result, the findings largely reflect the experiences of female, White British, participants and may not capture how cultural context, structural barriers, or experiences of discrimination shape help-seeking and engagement with primary care for people from minority ethnic backgrounds. Lower participation from minority ethnic groups in UK health research has been widely documented and may reflect factors such as trust, previous experiences with services, and structural barriers [49]. Future studies may benefit from more collaborative recruitment approaches, for example by building relationships with voluntary and community organisations or faith groups to support participation from a wider and more diverse population.

A further limitation relates to recruitment through GP practices. While this approach was appropriate given the study’s focus on experiences of primary care, it may have limited participation to individuals already connected to GP services and excluded those engaging primarily with community or voluntary sector organisations. Interviews were conducted online, which may have excluded individuals experiencing digital exclusion, including those without access to suitable devices, reliable internet access or data availability, or the digital skills required to participate [50]. However, the online format also reduced travel and logistical demands and may have enabled participation for individuals who might otherwise have struggled to attend in-person interviews. In addition, as the study was advertised through GP practices and focused on experiences of care, participation may have been influenced by factors such as health literacy, confidence in engaging with research, and familiarity with healthcare systems, which may have shaped who felt able or motivated to take part.

Future studies could further explore ways of improving primary care experiences for people with CMHD, for example through participatory approaches such as focus groups that invite people with CMHD to identify priorities for care. Another under-researched area is that of administrative and other support staff in primary care settings. Exploring their experiences of working within what can sometimes be a pressured environment could bring helpful insights into barriers to delivering accessible care.

## Implications and conclusions

There is a lack of qualitative research into the experiences of people with CMHD as understood by MIND [1] and the Community Mental Health Framework for Adults and Older Adults [2]. With the continued expansion of the framework in England, this is a timely piece of research. These findings provide timely insights into how people with CMHD experience and navigate primary care. Although grounded in the English Community Mental Health Framework, these findings have wider relevance for countries seeking to strengthen community-based mental health care and the role of primary care in supporting people with complex needs.

These findings add weight to previous research that has called for person-centred and flexible collaborative care with a focus on connection [18, 51–53]. Many of the participants expressed a preference for continuity of care to enable them to build up a relationship with their clinician. Introducing more flexible care, allowing reasonable adjustments for service users and simpler booking procedures, would reduce the onus of responsibility and effort from service users. As GPs are the ‘gatekeepers’ to additional support, trying to develop this continuity of care in GP practices would be beneficial.

There are now many health professionals attached to GP surgeries, so there is an opportunity to draw on a wide range of skills for this purpose. The benefit of continuity of care is that clinicians can notice the signs that someone may be struggling. In this context, many of the participants found it difficult to express their worries or needs. A trauma informed service that recognises trauma exposure as a common experience of those with CMHD may help clinicians prioritise safety, empathy, and collaboration within consultations, supporting more trusting relationships. Future research could explore how relational aspects of primary care influence engagement with services for people living with complex mental health difficulties.

People living with CMHD described primary care as a crucial but often difficult point of access to support. Their accounts highlight the damaging effects of diagnostic uncertainty, fragmented care, inflexible services, and responses that were experienced as dismissive or inadequate. At the same time, participants showed considerable insight, agency, and determination in trying to understand and manage their distress. The findings suggest that primary care for people with CMHD may be improved through continuity of care, collaborative and honest communication, recognition of patients’ own expertise, and more flexible pathways to support. Greater attention to these relational and organisational aspects of care may help reduce experiences of abandonment and improve engagement with services.

While many of the challenges described by participants—such as diagnostic uncertainty, barriers to accessing services, and the importance of continuity and communication—are already recognised in primary care, our findings suggest that these factors interact in ways that are less often acknowledged. Participants’ difficulties did not arise from a single barrier but from the cumulative effects of fragmented services, rigid organisational processes, and symptoms that themselves hindered help-seeking. Understanding these experiences, in research and service design, requires a systems perspective that recognises how patient needs, clinical relationships, and service structures interact [54]. Addressing the needs of people with CMHD may therefore require not only improvements in individual clinical encounters but also greater attention to how primary care systems are organised and how relational continuity, flexibility, and patient expertise are supported within them.

## Supplementary Information

Supplementary Material 1.

## Data Availability

The datasets generated and/or analysed during the current study are not publicly available in an open repository due to ethical restrictions related to participant confidentiality. However, participants provided informed consent for their data to be accessed and used by other authorised researchers, provided that confidentiality is preserved in accordance with the consent agreement. Accordingly, the data may be made available to qualified researchers upon reasonable request, subject to appropriate ethical approval and data use agreements.
