# Supplementary material for: Experiences of people with complex mental health difficulties accessing help from primary care services: a qualitative interview study
Source: BMC Prim Care. 2026 May 6;27:246. doi: 10.1186/s12875-026-03322-5 (PMC13321680; doi:10.1186/s12875-026-03322-5)
Supplement: Supplementary file 1 — Supplementary Material 1. [file 12875_2026_3322_MOESM1_ESM.docx]

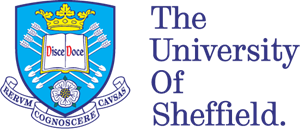


**Interview Topic Guide (for patients)**

**Version 1.1; 30/06/22**

**Understanding Services for people with Complex Mental Health Difficulties**

**(UNSEEN)**

| ---RESEARCH BACKGROUND--- |
| --- |
| This work aims to narrow the gaps between people with complex mental health difficulties, their GPs and specialist services. Complex mental health difficulties include (but are not limited to) personality disorders, neurodevelopmental co-morbidities, and the consequences of trauma. Complex mental health difficulties are increasingly recognised in NHS policy as important. |
| ---RESEARCH OBJECTIVES--- |
| The overarching research question is “How can general practices better identify people with complex mental health difficulties and provide care in a way which integrates primary care and specialist services?”.  **Aims:** To understand how people with complex mental health difficulties regard their conditions and how they experience primary care and its interaction with specialist mental health services. |

---DISCUSSION GUIDE BEGINS---

| ---OVERVIEW OF INTERVIEW FLOW--- | | |
| --- | --- | --- |
| 1 | **Introduction** | 3 MIN |
|  | **Objective:** To warm-up participants, introduce them to the interview process, and gather initial information about their role.  **Interviewer Introduction:**  Name of interviewer  Introduce the purpose of the study  Reassurance on confidentiality and consent to record the conversation.   - To start, can you tell me a little bit about yourself? - Probe: Age, Family, Occupation and/or Key responsibilities |  |
| 2 | **Perception of CMH** | 10 MIN |
|  | **Objective:** To examine the ways people with complex mental health currently describe, understand, and make sense of their problems.  **Possible questions:**   - Can you share with me some experiences of your mental health difficulties?   Probe: How have you made sense of this or how do you understand this |  |
| 3 | **Opinions on collaboration** | 10 MIN |
|  | **Objective:** To gather examples from the patient’s perspective of constructive working with primary care, particularly in relation to integration with community mental health services.  **Possible questions:**   - Thinking about your mental health condition/difficulty, have you sought any form of support for it?   If Yes: Where they chose support from and why that place/person was chosen.  If No: Why did they decide against it? What is the benefit of this to you?   - Have you had any support from your GP or some other primary care providers?   Probe: Was this positive or negative?   - Can you share this experience of poor care or support from your primary care provider with me? - Have you experienced any transition between primary to secondary care or vice versa? Can you share how this experience was for you with me? |  |
| 4 | **Opinions on existing & future generations** | 10 MIN |
|  | **Objective:** To understand ways in which people with complex mental health difficulties think of intergenerational mental health issues and how primary care teams should address these.  **Possible Questions:**   - How, if at all, do you feel your mental health difficulties has affected your family or the people around you in anyway?   If No: How have you managed to separate it/ detach your mental health difficulties and your social life?   - Do you think your mental health difficulties may influence your grandchildren in anyway?   If Yes: How do you rationalise it? How does it make you feel?  If No: How do you justify it?  Probe: Impact on Carers, impact on children, impact on family & friends  **Thank you for your answers so far- we are nearly at the end of this conversation. Now we would like to look towards the future.**  **Possible Question:**   - In an ideal world, if there weren’t the current issues you stated earlier, what do you think GP’s, or your GP could do better for you and people like you. - Probe: Continuity, Time, Wellbeing-checks, Effective collaborations |  |
| 5 | **Summing-up** | 2-3 MIN |
|  | **Objective:** To summarise and close discussion   - Interviewer to summarise and reiterate important points and interesting insights gathered from the discussion. - Any further points to add? - Thank you for your time and for helping us out with our study. |  |
